# One Gene, Many Facets: Multiple Immune Pathway Dysregulation in SOCS1 Haploinsufficiency

**DOI:** 10.3389/fimmu.2021.680334

**Published:** 2021-08-05

**Authors:** Julia Körholz, Anastasia Gabrielyan, John M. Sowerby, Felix Boschann, Lan-Sun Chen, Diana Paul, David Brandt, Janina Kleymann, Martin Kolditz, Nicole Toepfner, Ralf Knöfler, Eva-Maria Jacobsen, Christine Wolf, Karsten Conrad, Nadja Röber, Min Ae Lee-Kirsch, Kenneth G. C. Smith, Stefan Mundlos, Reinhard Berner, Alexander H. Dalpke, Catharina Schuetz, William Rae

**Affiliations:** ^1^Department of Pediatrics, University Hospital and Medical Faculty Carl-Gustav-Carus, Technische Universität Dresden, Dresden, Germany; ^2^UniversitätsCentrum für seltene Erkrankungen, Medizinische Fakultät Carl-Gustav-Carus, Technische Universität Dresden, Dresden, Germany; ^3^Cambridge Institute of Therapeutic Immunology and Infectious Disease, Jeffrey Cheah Biomedical Centre, University of Cambridge, Cambridge, United Kingdom; ^4^Department of Medicine, University of Cambridge, Cambridge, United Kingdom; ^5^Institute of Medical Genetics and Human Genetics, Charité - Universitätsmedizin Berlin, Corporate Member of Freie Universität Berlin, Humboldt-Universität zu Berlin, and Berlin Institute of Health, Berlin, Germany; ^6^Institute of Medical Microbiology and Virology, Medical Faculty Carl-Gustav-Carus, Technische Universität Dresden, Dresden, Germany; ^7^Department of Internal Medicine, Pneumology, Medizinische Fakultät Carl Gustav Carus, Technische Universität Dresden, Dresden, Germany; ^8^Department of Pediatrics, University Medical Center Ulm, Ulm, Germany; ^9^Institute of Immunology, Medizinische Fakultät Carl Gustav Carus, Technische Universität Dresden, Dresden, Germany; ^10^Max Planck Institute for Molecular Genetics, Research Group (RG) Development and Disease, Berlin, Germany; ^11^Berlin-Brandenburg Center for Regenerative Therapies, Charité - Universitätsmedizin Berlin, Freie Universität Berlin, Humboldt-Universität zu Berlin, and Berlin Institute of Health, Berlin, Germany

**Keywords:** inborn error of immunity (IEI), *SOCS1*, Hyper-IgE syndrome, autoimmunity, genetic pleiotropy

## Abstract

**Background:**

Inborn errors of immunity (IEI) present with a large phenotypic spectrum of disease, which can pose diagnostic and therapeutic challenges. Suppressor of cytokine signaling 1 (SOCS1) is a key negative regulator of cytokine signaling, and has recently been associated with a novel IEI. Of patients described to date, it is apparent that *SOCS1* haploinsufficiency has a pleiotropic effect in humans.

**Objective:**

We sought to investigate whether dysregulation of immune pathways, in addition to STAT1, play a role in the broad clinical manifestations of *SOCS1* haploinsufficiency.

**Methods:**

We assessed impacts of reduced *SOCS1* expression across multiple immune cell pathways utilizing patient cells and CRISPR/Cas9 edited primary human T cells.

**Results:**

*SOCS1* haploinsufficiency phenotypes straddled across the International Union of Immunological Societies classifications of IEI. We found that reduced SOCS1 expression led to dysregulation of multiple intracellular pathways in immune cells. STAT1 phosphorylation is enhanced, comparably with STAT1 gain-of-function mutations, and STAT3 phosphorylation is similarly reduced with concurrent reduction of Th17 cells. Furthermore, reduced SOCS1 E3 ligase function was associated with increased FAK1 in immune cells, and increased AKT and p70 ribosomal protein S6 kinase phosphorylation. We also found Toll-like receptor responses are increased in *SOCS1* haploinsufficiency patients.

**Conclusions:**

*SOCS1* haploinsufficiency is a pleiotropic monogenic IEI. Dysregulation of multiple immune cell pathways may explain the variable clinical phenotype associated with this new condition. Knowledge of these additional dysregulated immune pathways is important when considering the optimum management for *SOCS1* haploinsufficient patients.

## Introduction

Inborn errors of immunity (IEI) encompass rare disorders with a large phenotypic spectrum. They are increasingly recognized to display genetic heterogeneity, pleiotropy and incomplete penetrance (1, 2). This can make the diagnosis and interpretation of genetic results challenging. To help address these issues, the International Union of Immunological Societies (IUIS) proposes a structured approach to classify IEIs into nine groups based on the predominant clinical and immunological phenotype (2, 3). A genetic diagnosis is important as it can facilitate tailored management and personalized therapeutic strategies (4). Data from patient registries demonstrate a monogenic diagnosis in one third of European IEI patients (5, 6). This proportion is likely to increase further with the expanding number of monogenic IEIs described and increasing genetic testing (7).

Haploinsufficiency of *SOCS1*, the gene of suppressor of cytokine signaling (SOCS) 1, was recently identified as an autosomal dominant monogenic IEI (8–10). The SOCS protein family consists of eight members, SOCS 1-7 and the cytokine-inducible SH2-containing protein (CIS), each characterized by the presence of an SH2 domain and C-terminal SOCS box domain (11). SOCS proteins function as important negative regulators of cytokine signaling, and thus modulate cell functions. SOCS proteins exert their action by interacting with Janus kinases (JAKs) and non-receptor tyrosine kinase-2 (TYK2), and surface cytokine receptors, with varying affinities (11). SOCS1 binds and inhibits the phosphorylation of JAK1/2 and TYK2 (12) thus acting as an intracellular regulator.

**Figure 1:**
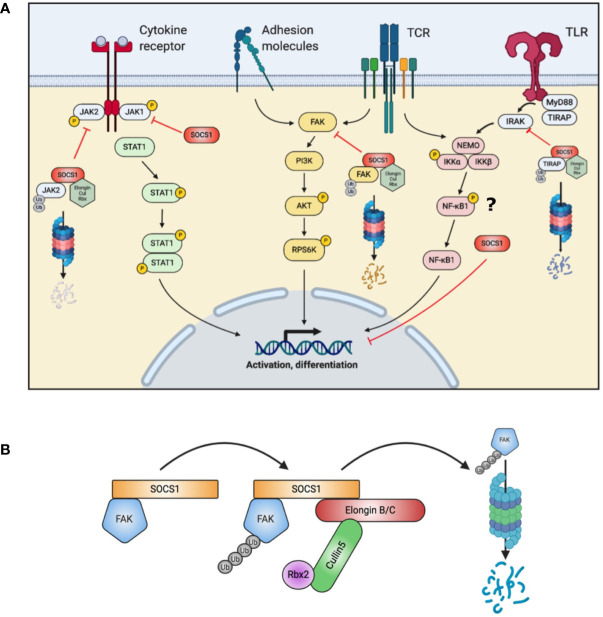
Summary of SOCS1 actions in T cells. **(A)** SOCS1 acts on multiple intracellular pathways, both by directly binding and blocking a target (JAK, NF-κB1) and as a substrate for the formation of an E3 ubiquitin ligase to target proteins for proteasomal degradation (FAK, TIRAP). **(B)** Simplified schematic of SOCS1 E3 ligase complex assembly with FAK (Focal adhesion kinase) accomplishing ubiquitination of signaling intermediates. AKT, Protein kinase B; Cul, Cullin; FAK, Focal adhesion kinase; IKK, inhibitor of nuclear factor kappa-B kinase; IRAK, Interleukin-1 receptor associated kinase; JAK, Janus kinase; MyD88, Myeloid differentiation primary response 88; NEMO, NF-kappa-B essential modulator; NFκB, nuclear factor kappa-light-chain enhancer of activated B cells; PI3K, Phosphoinositide 3 Kinase; Rbx, RING-box protein; RPS6K, Ribosomal protein s6 kinase; SOCS, suppressor of cytokine signaling; STAT, signal transducer and activator of transcription; TCR, T cell receptor; TIRAP, toll-interleukin 1 receptor (TIR) domain containing adaptor protein; TLR, Toll-like receptor; Ub, Ubiquitin.

Apart from this particular function of SOCS1, other investigations suppose that *SOCS1* impacts on multiple immune pathways beyond enhanced signal transducer and activator of transcription (STAT) 1 phosphorylation (13–15) (**Figure 1A** **)**. SOCS1 additionally functions as an E3 ligase substrate (16): SOCS1 binds to ElonginB/C *via* interaction of the C-terminal SOCS-box domain, to form a Cullin5 Rbx2 E3 ligase complex (12) (**Figure 1B**). This complex ubiquitinates target proteins for proteasomal degradation (12). One such protein is focal adhesion kinase (FAK) 1, encoded by *PTK2 (* 15). FAK1 is a ubiquitously expressed non-receptor protein tyrosine kinase which participates in a phosphorylation signaling pathway downstream of the T-cell receptor, cytokine receptors and adhesion molecules (17). FAK1 may activate the AKT – p70 ribosomal protein S6 kinase (RPS6K) β1 pathway. Another target for the SOCS1 E3 ligase is Toll/interleukin-1 receptor domain-containing adaptor protein (TIRAP), which is an important adaptor protein involved in Toll-like receptor (TLR) responses (18).

**Figure 2:**
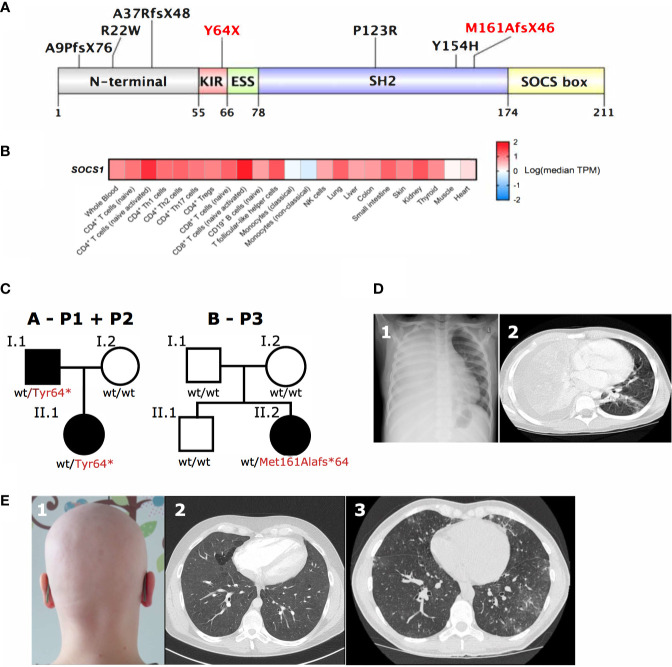
SOCS1 tissue expression, genetic and clinical pleiotropy. **(A)** Schematic of SOCS1 protein showing locations of pathogenic mutations described. Mutations further investigated here highlighted in red. KIR, Kinase inhibitory region; ESS, Extended SH2 subdomain; SH2, SH2 domain; SOCS box, SOCS box domain (Uniprot O15524). **(B)** Heatmap of SOCS1 expression across immune cells and tissues. TPM, transcripts per million. **(C)** Family trees of two *SOCS1* ^+/-^ kindreds with a total of three affected individuals. **(D)**_1_Chest radiography and _2_computed tomography (CT) scan showing dense consolidation of the entire right lung field with associated empyema due to bacterial infection in P1 with *SOCS1* ^+/-^. **(E)**_1_Autoimmune alopecia totalis in the same patient (P1) as well as _2_CT scans of widespread ground glass changes (P2) and _3_granulomatous lymphocytic interstitial lung disease next to bronchiectasis (P3) in two unrelated patients with *SOCS1* ^+/-^ (P2, P3).

Although only a limited number of *SOCS1^+/-^* individuals have been identified to date (8–10), it is already apparent that they show highly variable clinical phenotypes (**Table 1**). Reported mutations in *SOCS1* are monoallelic, resulting in loss-of-function of a single allele. Frameshift, truncating and missense mutations have been identified in three of the five protein domains of SOCS1 (8–10) (**Figure 2A**). However, clinical phenotypes lie across different IUIS groups: immune dysregulation with multisystem autoimmunity, autoimmune disease such as systemic lupus erythematosus (SLE), chronic autoimmune cytopenias and malignancy have all been documented (8–10). To investigate how these distinct clinical presentations may arise, we studied the role of SOCS1 in *SOCS1* ^+/-^ patient immune cells and CRISPR/Cas9- edited primary human T cells.

**Table 1:** Clinical phenotypes of *SOCS1^+/-^* patients.

Phenotype	This report and Thaventhiran et al. **(** 8 **)**			Lee et al. **(** 9 **)**		Hadjadj et al. **(** 10 **)**				
	HIES, severe infections	Severe Atopy, Hyperinflammation	CVID, GLILD, bacterial infections	Autoimmunity	Multisystem autoimmunity	Autoimmunity, infections	Autoimmunity	Autoimmunity, SLE	Autoimmunity	Autoimmunity, Malignancy
**Sex**	**Female**	**Male**	**Female**	Male	Male	Female	Female	Male	Female	Male
	**P1**	**P2**	**P3**							
**Age at first symptom**	**5 yrs**	**7 yrs**	**8 yrs**	2yrs	14yrs	5 mo	44yrs	16yrs	6yrs	3yrs
**Mutation**	c.(192C>G); p.(Tyr64*)	c.(192C>G); p.(Tyr64*)	c.(480_481insGCGGC); p.(Met161Alafs*46)	c.(106del); p.(Ala37Argfs*48)	c.(24del); p.(Ala9Profs*76)	c.(24delA); p.(Ala9fs*76)	c.(460T>C); p.(Tyr154His)	c.(64C>T); p.(Arg22Trp)	c.(368C>G); p.(Pro123Arg)	c.(24delA); p.(Ala9fs*76)
**Location in SOCS1**	**KIR domain**	**KIR domain**	**SH2 domain**	N-terminal	N-terminal	N-terminal	SH2 domain	N-terminal	SH2 domain	N-terminal
**Infections**	**Pneumonia with empyema, dental abscesses**	**URI**	**URI, Pneumonia**	Otitis media		URI shingles	–	–	–	–
			**Otitis media**							
	**UTI, local HSV infections, shingles**									
**Pathogen**	***S. pneumoniae*, HSV1, VZV**		***S. pneumoniae***		Sars-CoV2	VZV	–	–	–	–
			***Moraxella catarrhalis***							
**Autoimmunity**	**Neutropenia, AIHA, chronic ITP, Alopecia totalis**	**Hashimoto thyroiditis**	**ITP, AI-Hepatitis**	AIN, ITP, AIHA	AIN, ITP, AIHA	Neutropenia, ITP, AIHA	AI-Hepatitis, AI-Pancreatitis	SLE (cut., LN)	ITP, Thyroiditis	Celiac disease
**Auto-Antibodies**	**Coombs**	**anti-PCA**	**Coombs**	anti-Neutrophil, anti-Erythrocyte warm-AB	anti-erythrocyte	Coombs	ANA, anti-SCL	ANA, anti-DNA, anti-SSA/anti-SSB	ANA, ant-TPO, anti-mitochondrial-M2, antiGP210	NA
	**anti-Cw, anti-IIb/IIIa, anti-Ia/IIa, anti-Ib/IX, GP2-AK**	**anti-H+/K+-**								
		**ATPase**								
		**anti-MPO**								
		**anti-MCV**								
		**anti-TPO**								
		**anti-PEA**								
		**anti- Citrullin, anti-Purkinje cell**								
**Autoinflammation**	**Atopic eczema, allergic rhino-conjunctivitis, allergic asthma, HSM**	**Allergic rhinoconjunctivitisallergic asthma**	**Splenomegaly**	Diarrhea, oral ulcers	MIS-C	Splenomegaly, lymphadenopathy	Psoriasis, spondyloarthritis	–	Polyarthritis	Psoriasis
			**GLILD**							
		**splenomegaly, pernicious anemia, EAA resulting in OP**	**chronic granulomatous uveitis**							
**Other, including malignancy**	**-**	**-**	**-**	–	–	–	–	Growth-hormone deficiency	–	Hodgkin lymphoma
**Therapies**	**Prednisolone**	**Prednisolone**	**Prednisolone, Rituximab**	Corticosteroids, MMF	Corticosteroids	Corticosteroids, Rapamycine	MTX, anti-TNFa biologics	Corticosteroids, Hydroxychloroquine, MMF	Corticosteroids, Hormonal substitution	Chemotherapy
		**Levothyroxine, Vitamin B12**			MMF					
	**IVIG**									
	**Romiplostim**									
			**IVIG**		IVIG					
					Eltrombopag					
						IVIG				

*Stop codon.

SOCS1 is expressed in both hematopoietic and non-hematopoietic cells (19, 20) (**Figure 2B**), which has important implications if considering hematopoietic stem cell transplantation as curative option in IEI patients. Additionally multiple effects of SOCS1 on intracellular immune pathways are important to consider when evaluating potential (targeted) treatments in *SOCS1* ^+/-^ patients.

## Methods

### Patients, Controls, and PBMC Isolation

Patients and healthy controls were enrolled on protocols approved by ethics review committees (Dresden: EK 464122017; Cambridge: 13/EE/0305), and provided written informed consent. Patient and healthy donor peripheral blood mononuclear cells (PBMC) were isolated by density-gradient centrifugation using Biocoll separating solution (density 1.077 g/ml, Biocell). PBMC were resuspended in RPMI culture media (Gibco), supplemented with glutamate (100m, Life Technologies), penicillin/streptomycin (100 U/100μg/ml, Life Technologies) and 10% fetal bovine serum (PAN Biotech).

### PBMC Stimulation and FACS Analysis

Intracellular pSTAT1 and total STAT1 kinetics were determined by FACS analysis. 50 µl whole blood was stimulated according to BD Phosflow™ T Cell Activation Kit Instruction Manual in polystyrene round-bottom tubes (Becton Dickinson Falcon). Blood was stimulated with Imukin^®^ 100ng/ml for 0–180min at 37°C. Erythrocytes were lysed and leucocytes fixed with 1 ml of pre-warmed 1x lyse/fix buffer. The cells were then permeabilized by adding 300 µL of cold perm buffer III on ice for 30 min, stained with 750 µl stain buffer and incubated for 1 h in the dark at 4°C with following antibodies: anti-human CD14-FITC (BD Biosciences 345784), isotype STAT normal mouse IgG2a Alexa Fluor^®^ 647 (Santa Cruz Biotechnology sc-24637) and Alexa Fluor^®^ 647 mouse Anti-Stat1 (pY701) (BD Biosciences 612597). For Th1 and Th17 percentages PBMCs were stained with CD3 – BV605 (Biolegend), CD4 - APC-eFluor780 (eBioscience), CD8 – BV650 (eBioscience), CXCR3 – FITC (BioLegend), CCR6 – PE-Cy7 (BioLegend), CD45RA – PerCP (eBioscience) GZMB - e450 (BioLegend), CFSE - Invitrogen. All data were collected with LSR II (Becton Dickinson) and analyzed with FlowJo software (Treestar, Ashland, OR, USA).

### PBMC Stimulation and Cytokine Measurement

PBMC (1x10^6^/ml) were left unstimulated or stimulated with 10 ng/ml PMA (Phorbol 12-myristate 13-acetate) and 1µg/ml Calcium-Ionophore (both Merck KGaA, Darmstadt, Germany) under addition Brefeldin A (1µl/ml, BD-Biosciences San José, CA) of for 12h overnight. Cells then were harvested and washed twice with PBS/1%FCS. For surface staining, cells were incubated with anti-CD45RO-PE (5µl/test) (Biolegend, San Diego, CA), anti-CD3-APC (2µl/test), anti-CD4-APC-AlexaFluor750 (5µl/test) and anti-CD45-Krome Orange (5µl/test) (Beckman Coulter, Krefeld, Germany) for 30 min at 4°C. After being washed twice with PBS/1% FCS, cells were fixed and permeabilized with 100µl Cytofix/Cytoperm™ (BD Biosciences, San José, CA) for 20 min at 4°C and then washed twice according to the manufacturer’s instructions. For intracellular staining, cells were incubated with anti-IFNγ-FITC (5µl/test), anti-IL4-PE Cy7 (5µl/test) (both Biolegend) and anti-IL-17A eFlour 450 (5µl/test) (eBioscience/Thermo Scientific) and anti-CD4 APC-AlexaFluor750 (2µl/test) (Beckman-Coulter, Krefeld, Germany) for 45min at 4°C. Cells were washed twice with Perm/Wash Buffer (BD Biosciences) and diluted in 500µl PBS/1% FCS. Analysis was performed on a Navios Flow-Cytometer (Beckman-Coulter-Krefeld). For measurement of IFN-*γ* and IL-4 production 50,000 T cells per 96 well were stimulated with anti-CD3/CD28 beads (Miltenyi Biotec) (ratio cells:beads 1:1 for 24 hours. Supernatants were then measured using the Meso Scale Discovery V-PLEX Proinflammatory Panel 1 Human Kit (cat# K15049D).

### PBMC Stimulation and Immunoblotting

1x10^6^ PBMCs/ml were incubated in 24 well- plates with 50 ng/ml of recombinant human IFN-γ (PeproTech) for 0 (at rest), 30, 120 and 180 min in complete RPMI medium. To ensure equal loading, protein concentrations were assessed *via* Pierce™ BCA Protein Assay Kit (Thermo Scientific). Lysates were denatured in 1x LDS buffer (Invitrogen) then boiled for 10 mins at 70°C. Lysates were then run on NuPAGE Novex 4-12% gels (Invitrogen) with Spectra Multicolour Broad Range Protein Ladder (Invitrogen). Electrophoresis was run a 350A for 1 hour to transfer protein to a PDVF membrane. Membranes were then blocked with 5% non-fat milk in 1x Tris buffered saline with 0.1% Tween20 (Sigma) (TBST) for 1 hour at room temperature. Primary antibodies used were B-actin, Phospho-STAT1, STAT1, pJAK1, STAT3, pSTAT3, Cyclophilin B, NFKB1, pAKT, AKT SOCS1, FAK, and JAK2 (all Cell Signaling). Membranes were incubated overnight with primary antibodies, then washed 3x 10mins with TBST, before being incubated with a rabbit HRP-conjugated secondary antibody for 1 hours at room temperature. Membranes were washed a further 3x 10mins with TBST then developed with chemiluminescence ECL or Femto (Thermo Fisher).

### Molecular Genetics Analysis

Genomic DNA (index case and parents) was isolated from peripheral blood and enriched with a SureSelect Human All Exon Kit V6 (Agilent technologies) for subsequent Trio-WES on the Illumina system. Reads were aligned to human genome build GRCh37/hg19. Sequence reads were called and analyzed according to an in-house standard operating procedure using the VarFish platform (21). Variant filtration included filtering by minor allele frequency, mode of inheritance, functional prediction and constraint metrics, such as LOEUF score for LOF-variants (22). Candidate variants were confirmed by Sanger sequencing. The SOCS1 variants have been deposited in ClinVar under accession numbers VCV000977214 and SCV001653515.1.

### Measurement of Interferon Signature

Total RNA was extracted from PBMCs using the RNeasyMini Kit (Qiagen) followed by DNase I digestion. Gene expression was determined by quantitative real-time RT-PCR using Taqman Universal PCRMaster Mix (Applied Biosystems) on an ABI7300 and normalized to the expression of glyceraldehyde-3-phosphate dehydrogenase and hypoxanthine phosphoribosyltransferase 1 (Hs02800695_m1). For calibration, a calibrator cDNA was included in each assay. Target genes were analyzed using predesigned TaqMan probes for IFI44 (Hs00951349_m1), IFI44L (Hs00915292_m1), IFIT1 (Hs01675197_m1), ISG15 (Hs01921425_s1), RSAD2 (Hs01057264_m1), and SIGLEC1 (Hs00988063_m1). Oligonucleotides used for quantitative RT-PCR of GAPDH were for-GAAGGTGAAGGTCGGAGTC, rev-GAAGATGGTGATGGGATTTC, and FAM-CAAGCTTCCCGTTCTCAGCC-TAMRA. The IFN score was calculated as previously described by Wolf et al. (23).

### Magnetic Cell Separation

Human blood from healthy controls or SOCS-1 haploinsufficient patients was collected and fractionated for peripheral blood mononuclear cells (PBMC) by Pancoll (P04-60500, PAN-Biotech). PBMC were proceeded with human Pan Monocyte Isolation Kit (130-096-537, Miltenyi Biotec) following manufacture’s procedure. Magnetic microbeads-stained PBMC were subjected to autoMACS Pro Sparator (Miltenyi Biotec) for un-touched monocyte enrichment. 50,000 per well of sorted pan-monocytes were seeded into 96-well plate and cultured with RPMI-1640 medium (R2405-500ML, Sigma-Aldrich) + 5% Human AB serum (P30-2501, PAN-Biotech) for further stimulation.

### Pan-Monocytes Stimulation

Sorted pan-monocytes from healthy controls and SOCS-1 haploinsufficient patients were left untreated (UT), or stimulated with different TLR ligands. Briefly, TLR stimulants used were: Pam3CSK4 (tlrl-pms) TLR2, LPS (tlrl-peklps) TLR4, Flagellin (tlrl-pafla) TLR5, R837 (tlrl-imqs) TLR7, R848 (tlrl-r848) TLR7/8, TL8-506 (tlrl-tl8506) TLR8, and ODN 2216 (tlrl-2216) TLR9 were all from Invivogen. RIBOXXOL (A-00102, Riboxx) is a double-stranded RNA duplex which stimulates TLR3. Pan-monocytes were stimulated with RIBOXXOL at 2 ng/ml, LPS at 0.5 µg/ml, ODN 2216 at 1 µM, and Flagellin, R837, R848, TL8-506 all at 1 µg/ml for 20 hours.

### Cytokine Bead Arrays

Cytokine Bead Arrays (CBA) were performed by using LEGENDplex human anti-virus response panels 13-plex (740390, Biolegend). After stimulation, supernatants were collected by centrifugation at 800G for 5 minutes, and then subjected to CBA 13-plex panels following the manufacture’s procedure. Levels of IL-1β, IL-6, TNF-⍺, IP-10, IL-29(IFN-λ1), IL-8, IL-12p70, IFN-α2, IL-28A/B(IFN-λ2/3), GM-CSF, IFN-β, IL-10, and IFN-γ were measured simultaneously by MACSQuant analyzer 10 (Miltenyi Biotec). FCS files were further analyzed by LEGENDplex data analysis software version 8.0 (Biolegend).

### Cytokine Heatmap Hierarchical Clustering

Cytokine results were transformed by [log10(original cytokine concentration x) – row means (mean value of each cytokine after log10 transformation) of log10(x)]. Heatmap was constructed of row Z scores and then clustered by average linkage with Euclidean distance measurement method. Heatmap was generated using heatmap.2 in R studio.

### Autoantibody Testing

To look for the induction of different cell, organ and tissue specific autoantibodies (AAB) as well as ABs directed against highly conserved non-organ specific autoantigens (AAGs) we used a broad panel of AAB test methods (see **Table 1**). Different antigenic targets were used by indirect immunofluorescence tests (Euroimmun AG, Lübeck, Germany; GA Generic Assays, Dahlewitz, Germany) to identify non-organ specific AAB (HEp-2 cell assay: antinuclear antibodies, anticytoplacmic antibodies) (24), antineutrophil cytoplasmic antibodies (human neutrophile granulocytes), endomysial antibodies (primate intestine), AABs characteristic for autoimmune liver diseases (rat organ kryostat sections: LKM, AMA, SMA), AABs specific for autoimmune endocrine diseases (primate endocrine organ sections: adrenal gland, parathyroid gland, pancreas, ovary and testis sections), anti-glomerular basement antibodies (kidney sections) and AABs specific for kidney and autoimmune neurologic diseases (HEK293 cells specific transfected with target autoantigens like NMDA receptor). Furthermore, immunoassays for the detection of AABs directed against highly purified or recombinant target antigens such as line immunoassays (D-tec s.a., Moons, Belgium; GA Generic Assay, Dahlewitz, Germany; ravo Diagnostika GmbH, Freiburg, Germany), enzyme immunoassays (GA Generic Assays and Medipan GmbH, Dahlewitz, Germany; AESCU Group GmbH & Ko KG, Wendelsheim, Germany; Orgentec Diagnostika GmbH, Mainz, Germany), chemiluminescence immunoassay (Inova Diagnostics, Inc., San Diego, USA) and radioimmunoassays (DLD Diagnostika GmbH, Hamburg, Germany) were used to identify AABs specific for autoimmune liver (LKM-1, LC1, SLA, actin, gp210, sp100, GBM, MPO), neurologic (Ma1, Ma2, Amphiphysin, CV2, HuD, GAD65, SOX1, Zic4, DNER, AchR, MuSk, VGCC, VGKC, 10 different gangliosides), endocrine (IFA, H+K+-ATPase, GAD, IA2, INS, ZnT8, TG, TPO, 21H), kidney (GBM, MPO, PR3), gastrointestinal (GTG, GDP, ASCA, GP2) as well as systemic autoimmune diseases (MPO, PR3, CCP, MCV, CEP-1, RA33, RF, C1q, 5 different aPL).

### Primary Human CD4^+^ T Cell CRISPR/Cas9

Healthy control CD4^+^ T cells were isolated from fresh blood PBMCs using human CD4^+^ T cell isolation Kit (130-096-533, Miltenyi Biotec) according to the manufacturer’s instructions. 5x10^6^ CD4^+^ T cells were electroporated using a Nucleofector 2b program V024 (resting T cells) (Lonza). *SOCS1* crRNA guide: CGGCGUGCGAACGGAAUGUGGUUUUAGAGCUAUGCU was combined to form sgRNA heteroduplexes with Alt-R® CRISPR-Cas9 tracrRNA (1072534, Integrated DNA Technologies), then with Alt-R® CRISPR-Cas9 S.p. Cas9 D10A Nickase V3 (1081059, Integrated DNA Technologies) for electroporation. Negative control (Alt-R® CRISPR-Cas9 Negative Control crRNA). Following electroporation cells were rested for 48 hours in antibiotic free media before transfer to complete RPMI.

## Results

### Clinical Presentation and Immunophenotype Are Heterogenous in *SOCS1* Haploinsufficient Patients

*SOCS1* haploinsufficient patients are known to present with variable phenotypes with incomplete penetrance. *SOCS1* patients may present with Hyper IgE-like syndrome (HIES) with eczema and purulent infections, P1 had a HIES score of 48 (25) (**Table 1**, **Table S2** and **Figures 2C, D**), eosinophilic allergic alveolitis (EAA) in P2 (**Table 1** and **Figures 2C, E**) as well as common variable immunodeficiency (CVID)-like phenotype with hypogammaglobulinemia, T-cell lymphopenia and granulomatous lymphocytic interstitial lung disease (GLILD) in P3 (**Table 1**, **Table S1** and **Figures 2C, E**). Autoimmune and autoinflammatory features are frequent in almost all patients (**Table 1**, **Figure 2E**). These variable phenotypes with autoimmunity are evident from the overview of *SOCS1* haploinsufficiency phenotypes (**Table 1**) which demonstrates the clinical spectrum spans from autoimmunity to infections and malignancy.

Immunophenotyping results from *SOCS1* ^+/-^ patients are variable, with hypogammaglobulinemia and a B-cell-maturation deficiency described in one patient. T-cell counts and subpopulations were normal in one kindred, P3, then following steroid treatment for lung disease, showed reduced T cell numbers with normal subpopulations. T-cell proliferation in response to anti-CD3/CD28 stimulation was normal, as described previously (10), but we observed increased cell division in response to interleukin 7 in SOCS1 knockdown CD4^+^ T cells derived from healthy donors (**Figure S7**).

*SOCS1* haploinsufficiency impacts on intracellular JAK/STAT signaling of patient immune cells.

**Figure 3:**
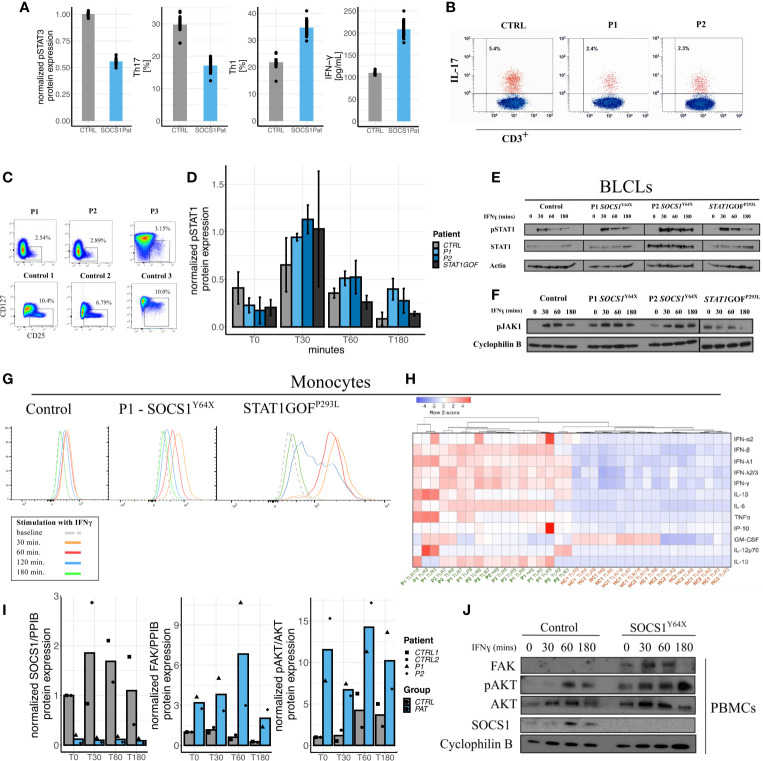
Impact of SOCS1 haploinsufficiency on immunophenotype, JAK/STAT signaling as compared to STAT^GOF^ and intracellular signaling pathways beyond JAK/STAT. **(A)** Reduced pSTAT3 in *SOCS1* ^+/-^ T cell blasts following IL-6 stimulation, reduced Th17 (CD3+CD4+CXCR3-CCR6+) cells in *SOCS1* ^+/-^patients, increased proportions of Th1 (CD3+CD4+CXCR3+CCR6-) cells in *SOCS1* ^+/-^ patients, and increased IFN-γ from CD4+ T cells in response to anti-CD3/CD28 stimulation and compared with healthy controls. **(B)** FACS plots of IL-17 expression in fresh T cells (CD3^+^) of P1 and P2 compared to a healthy control. **(C)** FACS plots of regulatory T cells (Tregs) from frozen PBMCs of P1 and P2 and fresh blood of P3 as well as three representative plots from healthy controls (published in **Supplementary Material** of 8). **(D)** Graphical expression of immunoblots of pSTAT1 from a time-course of IFN-γ stimulated PBMCs of *SOCS1* ^+/-^ patients and a *STAT1* ^GOF^ patient (p.(Pro293Leu), located in the Coiled coil domain of STAT1). Error bars: Mean±S.E.M. **(E)** Immunoblots of pSTAT1 and STAT1 from an IFN-γ stimulation time-course from BLCLs from *SOCS1* ^+/-^ and *STAT1* ^GOF^ patients. **(F)** Immunoblots of pJAK1 from an IFN-γ stimulation time-course from BLCLs from *SOCS1* ^+/-^ and *STAT1* ^GOF^ patients. **(G)** Mean fluorescence intensity time-course of pSTAT1 in monocytes from patients with *SOCS1* ^+/-^ and *STAT1* ^GOF^. **(H)** Heatmap and hierarchical clustering of normalized cytokine production [log10(original cytokine concentration x) – row means (mean value of each cytokine after log10 transformation) of log10(x)] in response to TLR ligands (TLR 2-5 and TLR 7-9) in monocytes of *SOCS1* ^+/-^ patients and healthy controls. **(I)** Quantifications of immunoblots of IFN-γ 20ng/ml stimulation time-course in PBMCs for SOCS1, FAK and pAKT in P1 and P2 compared to two matched healthy controls. **(J)** Representative immunoblot of IFN-γ stimulation time-course in PBMCs for FAK and AKT in a *SOCS1* ^+/-^ patient.

To investigate the clinical overlap of P1 with the Hyper IgE Syndrome as *STAT3* ^LOF^ we explored similarities and differences in cytokine signaling *via* the JAK/STAT pathway. In *SOCS1* ^+/-^ patients’ T cells we observed reduced STAT3 phosphorylation, and a decrease in Th17 cells as well as IL-17 production (**Figures 3A, B**). *SOCS1* ^+/-^ patients also exhibited enhanced Th1 polarization of CD4^+^ T cells and increased production of interferon-gamma (IFN*γ*) (**Figure 3A**). Th1*, IL-4 production, and regulatory T cells were also reduced in *SOCS1* ^+/-^ patients (**Figure S1** and **Figure 3C**).

Investigation of peripheral blood mononuclear cells (PBMCs), monocytes and B-lymphoblastoid cell lines (BLCLs) derived from *SOCS* ^+/-^ patients (P1, P2 and P3) showed increased STAT1 phosphorylation, reminiscent of *STAT1* gain-of-function (GOF) patients (**Figures 3D, E, G**). These observations could be demonstrated in western blots of PBMCs and BLCLs, as well as and in phospho-flow assays on Monocytes. pSTAT1 expression was not only increased but seemed also prolonged compared to healthy controls and *STAT1^GOF^* patients. In contrast to *STAT1^GOF^* patients, *SOCS1* ^+/-^ patients showed increased and prolonged JAK1 phosphorylation in BLCLs (**Figure 3F**), which is consistent with the expected impact of *SOCS1* ^+/-^, but distinct from *STAT1^GOF^*. However, although JAK/STAT signaling seems to be hyperactivated in *SOCS1* ^+/-^ patients comparable to *STAT1^GOF^*, a transcriptional type I interferon signature was not prominent in P1 and P2 (**Figure S2**). Thus, perturbations in JAK – STAT1 signaling are found in *SOCS1* haploinsufficient patients.

### *SOCS1* Haploinsufficiency Disrupts Proteasomal Degradation *via* E3 Ligase Functions

Apart from JAK/STAT signaling, SOCS1 is an important regulator in multiple other intracellular pathways (**Figure 1A**). We therefore investigated how *SOCS1* haploinsufficiency in humans influenced other intracellular signaling pathways. Patients’ PBMCs and T-cell blasts were investigated for protein expression in the FAK1-AKT-RPS6K pathway *via* western blots. In *SOCS1* ^+/-^ patient cells we found FAK1 levels to be increased along with enhanced phosphorylated AKT and RPS6K (data shown for PBMCs in **Figures 3I, J** **)**, consistent with an increased activity in the FAK1 – AKT – RPS6K pathway. To confirm these results, we analyzed protein expression in *SOCS1* knockdown CD4+ T cells. Similarly we could observe increased FAK and JAK2 expression as well as increased AKT phosphorylation in this healthy control CD4^+^ T cell SOCS1 knockdown model (**Figures S5**, **S6**).

In the next step, we investigated TLR responses in *SOCS* ^+/-^ patients’ monocytes. Notably, we observed enhanced cytokine responses to a variety of TLR ligands (TLR2-5 and TLR 7-9) in SOCS1^+/-^ patients (**Figure 3H**). Pre-incubation and activation of monocytes with IFN-*γ* further increased differences in cytokine production between *SOCS* ^+/-^ patients and healthy controls, consistent with the induction of a hyperactivated state (**Figure S8**).

## Discussion

*SOCS1* haploinsufficiency has recently been described as a pleiotropic IEI with variable penetrance (8–10). Overall, the presentation of human *SOCS1* ^+/-^ appears to be predominantly autoimmunity as opposed to infection (**Table 1**). Additionally, malignant disease such as Hodgkin’s lymphoma has been observed in one *SOCS1* ^+/-^ patient (10). According to the national cancer institute, somatic *SOCS1* mutations could be identified in several B cell malignancies (26). Thus in the broad phenotypic spectrum of *SOCS1* haploinsufficiency, malignancy seems to be another complication treating physicians have to be aware of.

Indeed, the phenotypic spectrum exceeds distinct characterizations of other monogenic IEIs characterized by the IUIS classification. Our observation of a Hyper IgE-like syndrome and severe atopy phenotype in the first kindred reported here has additional clinical overlaps with *STAT3* ^LOF^ patients. Similar to *STAT3* ^LOF^ patients (27), we could observe reduced pSTAT3 expression and reduced Th17 cells in *SOCS1* ^+/-^ patients. Complications like severe bacterial pneumonia, organizing pneumonia and allergic asthma may point towards a role of SOCS1 in local immunity, e.g. in lung tissue. In patients with severe asthma, decreased SOCS1 expression correlated with eosinophilia and Th2-driven inflammation (28). Furthermore, mice with a selective knockout of the nuclear localization sequence (NLS) of SOCS1 presented with low-grade pulmonary inflammation and an increase in Th2-type cytokines (13). In our patients Th2-type cytokines in CD4+ T cells were not elevated as compared to healthy controls, emphasizing the importance of local effects such as inflammation of SOCS1 in different tissues.

Multiple autoimmune manifestations are reminiscent of other IEIs such as *STAT1^GOF^*, a monogenic IEI characterized by a broad spectrum of infectious and non-infectious, mostly autoimmune phenotypes in addition to chronic mucocutaneous candidiasis (CMC) (29). Unlike in *STAT1* ^GOF^ and *STAT3^LOF^* patients, CMC has not been observed in *SOCS1* ^+/-^ patients although Th17 cells and IL-17 cytokine production in *SOCS1* ^+/-^ patients were reduced. Delimiting the immunological phenotype of *SOCS1* ^+/-^ patients from *STAT1* ^GOF^ patients, we could observe some immunological differences. Unlike in STAT1^GOF^ patients T-cell proliferation in response to anti-CD3/CD28 stimulation was normal in T cells of SOCS1^+/-^ patients. Additionally regulatory T cells are reduced in most *SOCS1* ^+/-^ patients, unlike in *STAT1^GOF^ (* 30). Moreover, type I IFN-signature was not elevated in our own *SOCS1* ^+/-^ patients. This stands in contrast to *STAT1^GOF^* patients (31) and may be due to lack of intrinsic overactivation of the STAT1-interferon pathway. However, triggers like infections may easily tip this balance, as reported in two previously reported *SOCS1* ^+/-^ patients with autoimmune cytopenia (9).

The SOCS1 protein with its subdomains acts not only on JAK/STAT signaling but is also an important regulator in other intracellular signaling pathways. As SOCS1 functions as an E3 ligase substrate it is also involved in FAK-AKT-RS6K and TLR signaling. We demonstrated that *SOCS1* ^+/-^ patient cells showed an increased activity in the FAK – AKT – RS6K pathway further explaining the accumulation of autoimmunity. Whilst reduced SOCS1 appears associated with increased FAK1, we observed a greater increase in P1 compared to P2 (**Figure 3I**). Although FAK expression has not been reported to be influenced by age, additional elements of the AKT pathway have been (32). Therefore in *SOCS1* haploinsufficient patients age, or the specific impact of variants, may influence FAK expression. Further investigation in *SOCS1* haploinsufficient patient over a range of ages and variants are warranted to confirm these observations.

Investigating TLR responses in *SOCS* ^+/-^ patients, we observed enhanced cytokine responses. This observation is supported by previous investigations which identified SOCS1 as an important target in the negative regulation of TCR signaling and which demonstrated that the ablation of SOCS1 in human T cells enhances T cell proliferation (33). These increased even further by stimulation with IFN-*γ* imitating an immune response in a hyperactivated state. This *in vitro* observation may mimic the observed infection-induced autoimmune cytopenias in *SOCS* ^+/-^ patients (9, 10). Disruption of intracellular signaling *via* SOCS1 in these multiple intracellular pathways may partially explain the spectrum of clinical diversity in *SOCS1^+/-^* patients.

Both PBMCs and T cell blasts of patients P1 and P2 with the variant Y64X showed reduced SOCS1 expression to below 50% of controls (**Figure 3J** and **Figure S4**), whilst expression of SOCS1 protein was still evident in Y64X patient derived BLCLs (**Figure S3**). Regulation of SOCS1 is achieved by several mechanisms *in vivo*, including by translation of an upstream open reading frame (uORF) from an alternative translation start site in the *SOCS1* mRNA (34, 35). The variant Y64X may still allow translation of this repressive uORF from the mutated SOCS1 allele which may, in certain cell types and states, result in repression of SOCS1 below 50%. Determining how, and if, this regulatory mechanism is effecting SOCS1 expression in patients with pathogenic SOCS1 variants will further elucidate this interested mechanism of translational regulation and how it may play a role in the clinical disease severity of SOCS1-related diseases.

In summary, reduced SOCS1 shows clinical overlaps with different monogenic IEIs, such as *STAT3^LOF^* and *STAT1^GOF^*. On a cellular level it is associated with increased JAK – STAT1 signaling, and reduction of the SOCS1 E3 ligase function, which may account for the dysregulation of multiple additional immune pathways. These multiple effects are important to consider when treating *SOCS1^+/-^* patients. JAK inhibitors have been shown *in vitro* to reduce exuberant STAT1 phosphorylation seen in *SOCS1* ^+/-^ patients (10). Potential therapeutic efficacy of JAK inhibitors warrants further clinical investigation, but may not fully suppress the additional hyperactive pathways present in *SOCS1* ^+/-^. Treatment of autoimmunity associated with *SOCS1* ^+/-^ may therefore require a broader immunosuppressive approach. Whilst hematopoietic stem cell transplantation may offer a curative treatment for severe *SOCS1* ^+/-^ disease to prevent organ damage through chronic infections and/or autoimmunity, its potential limitations need to be considered due to the non-hematopoietic expression of *SOCS1*. There is need for the collection and study of larger cohorts of *SOCS1* haploinsufficient patients to confirm and further study the observations we present here of the multiple immune pathway dysregulation evident in this patient group.

## Data Availability Statement

The original contributions presented in the study are included in the article/**Supplementary Material**. Further inquiries can be directed to the corresponding authors.

## Ethics Statement

The studies involving human participants were reviewed and approved by Ethikkommission TU Dresden. Written informed consent to participate in this study was provided by the participants’ legal guardian/next of kin. Written informed consent was obtained from the individual(s), and minor(s)’ legal guardian/next of kin, for the publication of any potentially identifiable images or data included in this article.

## Author Contributions

JKö, CS and WR designed the research and collected data. JKö, WR, AG and CS wrote the manuscript. AD, L-SC, KS, M-AL-K, SM, RB and FB contributed to the study design and revised the manuscript. JKö, CS, JKl, MK, DB, NT, and RK contributed to patients’ clinical care. CS and WR supervised experimental work and data analyses. WR, JS, AG, KC, NR, L-SC, E-MJ, CW and DP designed and performed experiments and analyzed data: e.g. genetic analyses (FB, SM), CRISPR/Cas9- editing of primary human T cells (WR, JS), interferon signatures (ML-K, CW), autoantibody studies (NR, KC), TLR/cytokine analyses (L-SC, AD), immunophenotyping (WR, E-MJ), STAT phosphorylation assays (WR, AG, DP). All authors contributed to the article and approved the submitted version.

## Funding

This work was supported by a Wellcome Trust PhD for Clinicians Fellowship (216382/Z/19/Z) to WR, Rosemarie-Germscheid Stiftung (Foundation) to CS and the Deutsche Forschungsgemeinschaft (German Research Foundation), grant 369799452/404459235 to M-AL-K.

## Conflict of Interest

The authors declare that the research was conducted in the absence of any commercial or financial relationships that could be construed as a potential conflict of interest.

## Publisher’s Note

All claims expressed in this article are solely those of the authors and do not necessarily represent those of their affiliated organizations, or those of the publisher, the editors and the reviewers. Any product that may be evaluated in this article, or claim that may be made by its manufacturer, is not guaranteed or endorsed by the publisher.

## References

[1] Gruber C Bogunovic D. Incomplete Penetrance in Primary Immunodeficiency: A Skeleton in the Closet. Hum Genet (2020) 139:74557. 10.1007/s00439-020-02131-9 PMC7275875 32067110

[2] Bousfiha A Jeddane L Picard C Al-Herz W Ailal F Chatila T. Human Inborn Errors of Immunity: 2019 Update of the IUIS Phenotypical Classification. J Clin Immunol (2020) 40:66–81. 10.1007/s10875-020-00758-x 32048120 PMC7082388

[3] Picard C Bobby Gaspar H Al-Herz W Bousfiha A Casanova J-L Chatila T. International Union of Immunological Societies: 2017 Primary Immunodeficiency Diseases Committee Report on Inborn Errors of Immunity. J Clin Immunol (2018) 38:96–128. 10.1007/s10875-017-0464-9 29226302 PMC5742601

[4] Lenardo M Lo B Lucas CL. Genomics of Immune Diseases and New Therapies. Annu Rev Immunol (2016) 34:121–49. 10.1146/annurev-immunol-041015-055620 PMC5736009 26735698

[5] Shillitoe B Bangs C Guzman D Gennery AR Longhurst HJ Slatter M. The United Kingdom Primary Immune Deficiency (UKPID) Registry 2012 to 2017. Clin Exp Immunol (2018) 192:284–91. 10.1111/cei.13125 PMC5980391 29878323

[6] El-Helou SM Biegner A-K Bode S Ehl SR Heeg M Maccari ME. The German National Registry of Primary Immunodeficiencies (2012–2017). Front Immunol (2019) 10:1272. 10.3389/fimmu.2019.01272 31379802 PMC6659583

[7] Meyts I Bosch B Bolze A Boisson B Itan Y Belkadi A. Exome and Genome Sequencing for Inborn Errors of Immunity. J Allergy Clin Immunol (2016) 138:957–69. 10.1016/j.jaci.2016.08.003 PMC5074686 27720020

[8] Thaventhiran JED Lango Allen H Burren OS Rae W Greene D Staples E. Whole-Genome Sequencing of a Sporadic Primary Immunodeficiency Cohort. Nature (2020) 583:90–5. 10.1038/s41586-020-2265-1 PMC7334047 32499645

[9] Lee PY Platt CD Weeks S Grace RF Maher G Gauthier K. Immune Dysregulation and Multisystem Inflammatory Syndrome in Children (MIS-C) in Individuals With Haploinsufficiency of SOCS1. J Allergy Clin Immunol (2020) 146(5):1194–200.e1. 10.1016/j.jaci.2020.07.033 PMC7445138 32853638

[10] Hadjadj J Castro CN Tusseau M Stolzenberg M-C Mazerolles F Aladjidi N. Early-Onset Autoimmunity Associated With SOCS1 Haploinsufficiency. Nat Commun (2020) 11:5341. 10.1038/s41467-020-18925-4 33087723 PMC7578789

[11] Yoshimura A Suzuki M Sakaguchi R Hanada T Yasukawa H. SOCS. Inflammation, and Autoimmunity. Front Immunol (2012) 3:20. 10.3389/fimmu.2012.00020 22566904 PMC3342034

[12] Liau NPD Laktyushin A Lucet IS Murphy JM Yao S Whitlock E. The Molecular Basis of JAK/STAT Inhibition by SOCS1. Nat Commun (2018) 9:1558. 10.1038/s41467-018-04013-1 29674694 PMC5908791

[13] Zimmer J Weitnauer M Boutin S Küblbeck G Thiele S Walker P. Nuclear Localization of Suppressor of Cytokine Signaling-1 Regulates Local Immunity in the Lung. Front Immunol (2016) 7:514. 10.3389/fimmu.2016.00514 27917175 PMC5114302

[14] Strebovsky J Walker P Lang R Dalpke AH. Suppressor of Cytokine Signaling 1 (SOCS1) Limits Nfkappab Signaling by Decreasing P65 Stability Within the Cell Nucleus. FASEB J Off Publ Fed Am Soc Exp Biol (2011) 25:863–74. 10.1096/fj.10-170597 21084693

[15] Liu E Côté J-F Vuori K. Negative Regulation of FAK Signaling by SOCS Proteins. EMBO J (2003) 22:5036–46. 10.1093/emboj/cdg503 PMC204486 14517242

[16] Liau NPD Laktyushin A Lucet IS Murphy JM Yao S Whitlock E. The Molecular Basis of JAK/STAT Inhibition by SOCS1. Nat Commun (2018) 9:1558. 10.1038/s41467-018-04013-1 PMC5908791 29674694

[17] Chapman NM Houtman JCD. Functions of the FAK Family Kinases in T Cells: Beyond Actin Cytoskeletal Rearrangement. Immunol Res (2014) 59:23–34. 10.1007/s12026-014-8527-y 24816556 PMC4125427

[18] Mansell A Smith R Doyle SL Gray P Fenner JE Crack PJ. Suppressor of Cytokine Signaling 1 Negatively Regulates Toll-Like Receptor Signaling by Mediating Mal Degradation. Nat Immunol (2006) 7:148–55. 10.1038/ni1299 16415872

[19] GTEx Consortium. The Gtex Consortium Atlas of Genetic Regulatory Effects Across Human Tissues. Science (2020) 369:1318–30. 10.1126/science.aaz1776 PMC7737656 32913098

[20] Schmiedel BJ Singh D Madrigal A Valdovino-Gonzalez AG White BM Zapardiel-Gonzalo J. Impact of Genetic Polymorphisms on Human Immune Cell Gene Expression. Cell (2018) 175:1701–15.e16. 10.1016/j.cell.2018.10.022 30449622 PMC6289654

[21] Holtgrewe M Stolpe O Nieminen M Mundlos S Knaus A Kornak U. Varfish: Comprehensive DNA Variant Analysis for Diagnostics and Research. Nucleic Acids Res (2020) 48:W162–9. 10.1093/nar/gkaa241 PMC7319464 32338743

[22] Karczewski KJ Francioli LC Tiao G Cummings BB Alföldi J Wang Q. The Mutational Constraint Spectrum Quantified From Variation in 141,456 Humans. Nature (2020) 581:434–43. 10.1038/s41586-020-2308-7 PMC7334197 32461654

[23] Wolf C Brück N Koss S Griep C Kirschfink M Palm-Beden K. Janus Kinase Inhibition in Complement Component 1 Deficiency. J Allergy Clin Immunol (2020) 146(6):1439–42.e5. 10.1016/j.jaci.2020.04.002 32325142

[24] Chan EKL Damoiseaux J Carballo OG Conrad K de Melo Cruvinel W Francescantonio PLC. Report of the First International Consensus on Standardized Nomenclature of Antinuclear Antibody Hep-2 Cell Patterns (ICAP) 2014-2015. Front Immunol (2015) 6:412. 10.3389/fimmu.2015.00412 26347739 PMC4542633

[25] Grimbacher B Schäffer AA Holland SM Davis J Gallin JI Malech HL. Genetic Linkage of Hyper-IgE Syndrome to Chromosome 4. Am J Hum Genet (1999) 65:735–44. 10.1086/302547 PMC1377980 10441580

[26] National Cancer Institute GDP. SOCS1. Available at: https://portal.gdc.cancer.gov/genes/ENSG00000185338 (Accessed February 25, 2021).

[27] Chandesris M-O Melki I Natividad A Puel A Fieschi C Yun L. Autosomal Dominant STAT3 Deficiency and Hyper-Ige Syndrome Molecular, Cellular, and Clinical Features From a French National Survey. Med (Baltimore) (2012) 91:e1–19. 10.1097/MD.0b013e31825f95b9 PMC3680355 22751495

[28] Doran E Choy DF Shikotra A Butler CA O’Rourke DM Johnston JA. Reduced Epithelial Suppressor of Cytokine Signalling 1 in Severe Eosinophilic Asthma. Eur Respir J (2016) 48:715–25. 10.1183/13993003.00400-2015 27338192

[29] Toubiana J Okada S Hiller J Oleastro M Lagos Gomez M Aldave Becerra JC. Heterozygous STAT1 Gain-of-Function Mutations Underlie an Unexpectedly Broad Clinical Phenotype. Blood (2016) 127:3154–64. 10.1182/blood-2015-11-679902 PMC4920021 27114460

[30] Uzel G Sampaio EP Lawrence MG Hsu AP Hackett M Dorsey MJ. Dominant Gain-of-Function STAT1 Mutations in FOXP3 Wild-Type Immune Dysregulation-Polyendocrinopathy-Enteropathy-X-Linked-Like Syndrome. J Allergy Clin Immunol (2013) 131:1611–23. 10.1016/j.jaci.2012.11.054 PMC3672257 23534974

[31] Okada S Asano T Moriya K Boisson-Dupuis S Kobayashi M Casanova J-L. Human STAT1 Gain-of-Function Heterozygous Mutations: Chronic Mucocutaneous Candidiasis and Type I Interferonopathy. J Clin Immunol (2020) 40:1065–81. 10.1007/s10875-020-00847-x PMC8561788 32852681

[32] Cao J-N Gollapudi S Sharman EH Jia Z Gupta S. Age-Related Alterations of Gene Expression Patterns in Human CD8+ T Cells. Aging Cell (2010) 9:19–31. 10.1111/j.1474-9726.2009.00534.x 19878143

[33] Shifrut E Carnevale J Tobin V Roth TL Woo JM Bui CT. Genome-Wide CRISPR Screens in Primary Human T Cells Reveal Key Regulators of Immune Function. Cell (2018) 175:1958–71.e15. 10.1016/j.cell.2018.10.024 30449619 PMC6689405

[34] Gregorieff A Pyronnet S Sonenberg N Veillette A. Regulation of SOCS-1 Expression by Translational Repression. J Biol Chem (2000) 275:21596–604. 10.1074/jbc.M910087199 10764816

[35] Schlüter G Boinska D Nieman-Seyde SC. Evidence for Translational Repression of the SOCS-1 Major Open Reading Frame by an Upstream Open Reading Frame. Biochem Biophys Res Commun (2000) 268:255–61. 10.1006/bbrc.2000.2109 10679190

